# Anatomy, Descriptive and Surgical

**Published:** 1902-05

**Authors:** 


ANATOMY, DESCRIPTIVE AND SURGICAL. By HENRY GRAY, F. R. S., Lecturer on Anatomy at St. George's Hospital, London. Thoroughly revised American from the fifteenth English edition. In one imperial 8vo volume of 1,246 pages, with 780 illustrations. Philadelphia and New York: Lea Brothers & Co. 1901. [Price, with illustrations in black, cloth, $5.50 net; leather, $6.50 net. Price, with illustrations in colors, cloth, $6.25 net; leather, $7.25, net.]

A revision of Gray's anatomy interests every student of medicine. It is a perennial favorite, and with good reason. Henry Gray was doubly a genius, being equally a born anatomist and a born teacher. His methods of presenting anatomical knowledge in text and picture were such a conspicuous and rational advance in his first edition that it instantly won the foremost place, which has never since been disputed.

No better evidence of this is needed than the announcement of a new revised American edition from the fifteenth English edition. No introduction of this work is necessary, it suffices simply to say, "A new edition of Gray," and every student of medicine in America, as well as in England, knows that the demand for Gray's anatomy is as persistent today as it was twenty years ago.

The foremost anatomists have been engaged in the many successive revisions, of which the present is perhaps the most thorough-going. Every page has been scrutinised and whole sections rewritten, notably those on the brain, spinal cord, nervous system and viscera. The magnificent and unique series of illustrations has been enriched with 231 new engravings, and the use of colors has been greatly increased.

The publishers are to be congratulated on the efficient way in which they have built the book, making it a first-class work in every respect, thereby keeping pace with the editions.

